# Geographical variation, socioeconomic inequalities of low birth weight, and its relationship with maternal dietary diversity: Insights from the maternal infant and young child nutrition programme in Bangladesh

**DOI:** 10.7189/jogh.14.04209

**Published:** 2024-10-11

**Authors:** Md. Tariqujjaman, Arifa F Tanha, Mahfuzur Rahman, Gobinda Karmakar, Mustafa Mahfuz, Md. M Hasan, Ahmed E Rahman, Anisuddin Ahmed, Shams E Arifeen, Tahmeed Ahmed, Haribondhu Sarma

**Affiliations:** 1 Nutrition Research Division, icddr,b, Dhaka, Bangladesh; 2 School of Health and Rehabilitation Sciences, The University of Queensland, Brisbane, Australia; 3 Maternal and Child Health Division, icddr,b, Dhaka, Bangladesh; 4 Poche Centre for Indigenous Health, The University of Queensland, Brisbane, Australia; 5 Global Health and Migration Unit, Department of Women’s and Children’s Health, Uppsala University, Uppsala, Sweden; 6 The National Centre for Epidemiology and Population Health, College of Health and Medicine, The Australian National University, Canberra, Australia

## Abstract

**Background:**

Globally, every year, 20 million neonates are born with weights below 2500 g and are considered low birth weight (LBW). About 90% of these births occur in low- and middle-income countries. Information regarding the geographical variation, socioeconomic inequalities of LBW neonates, and the relationship between maternal inadequate dietary diversity and LBW is limited in rural areas of Bangladesh. We aimed to explore the geographical disparities and socioeconomic inequalities in the prevalence of LBW and its association with inadequate maternal dietary diversity.

**Methods:**

We extracted data from a large-scale evaluation programme conducted as a part of the maternal infant and young child nutrition phase two in Bangladesh, implemented by BRAC. We used the concentration index (CIX) to measure the socioeconomic inequalities of LBW. We performed a cluster-adjusted multiple logistic regression analysis to determine the association between LBW and maternal dietary diversity.

**Results:**

A total of 4651 children aged <5 years with their mother’s information were included. The overall prevalence of LBW was 13.5%. About 16% of mothers living in the poorest wealth quintile gave birth to LBW babies, whereas 10% of mothers living in the richest households gave birth to LBW babies. The CIX exhibited LBW babies were more prevalent among the socioeconomically worst-off (poorest) group (CIX = −0.08), indicating mothers of the poorest households are vulnerable to giving birth to normal-weight babies. An adjusted multiple logistic regression model indicated that mothers with inadequate dietary diversity had higher odds (adjusted odds ratio (AOR) = 1.27; 95% confidence interval (CI) = 1.04, 1.54) of giving birth to LBW babies. Notably, in the interaction of mothers’ age and dietary diversity, we found that adolescent mothers (aged ≤ 19 years) with inadequate dietary diversity had 2.56 times (AOR = 2.56; 95% CI = 1.14, 5.76) higher odds of giving birth to LBW babies compared to adult mothers (aged >19 years) who consumed diversified foods.

**Conclusions:**

Intervention strategies for reducing LBW prevalence should target the poorest households. Also, interventions for improving the dietary diversity of adolescent pregnant mothers are expected to reduce the number of LBW babies from the rural areas of Bangladesh.

Low birth weight (LBW) is a major public health challenge globally, particularly in low- and middle-income countries (LMICs). The LBW newborns have a birth weight of less than 2500 g, regardless of gestational age, as defined by the World Health Organization (WHO) [1]. According to the WHO estimates, 15–20% of all babies worldwide are born with LBW [2]. The number is equivalent to more than 20 million births yearly, with five million dying annually [2]. The incidence of LBW varies significantly among regions and nations, with the highest incidence occurring in LMICs, which account for 91% of the total instances [3]. South Asia reports twice as many cases of LBW as sub-Saharan African countries [3]. Among South Asia, Bangladesh, India, and Pakistan account for the majority of the LBW babies [4]. The prevalence of LBW reduced from approximately 36% in 2004 to 23% in 2015 [5,6], as reported by the national LBW survey of Bangladesh.

The LBW has many immediate and long-term adverse effects on health and human capital [7]. Epidemiological study shows that LBW infants are 20 times more likely to die than infants born with normal weight [8]. In addition, LBW newborns have a higher risk of child morbidity, mortality, childhood stunting, and long-term physical and developmental ill health, including adult-onset chronic conditions like cardiovascular disease, which tend to be significant financial burdens on families, communities, and healthcare systems [9]. Aside from behavioural and psychological issues, LBW babies face the risk of developing cognitive deficiencies, motor delays, cerebral palsy, and other conditions [10]. Furthermore, it is an alarming indicator of probable neonatal deaths, a predictor of future risks for malnutrition in the child, and an indirect indicator of the mother’s health [11].

Worldwide attempts have been undertaken to uncover LBW’s aetiology and determine risk factors. However, these can be complex and vary by geographical region [5,11–15]. Prior findings from studies across developed and developing nations imply that potential risk factors for LBW include a history of preterm birth [11], a mother giving birth at a younger age [12,13], inadequate prenatal care [5], underweight mother [12], shorter gestation interval [12], hard work and low nutritious food consumption during pregnancy [12,13], antepartum haemorrhage and anaemia [14], hypertension disorder and diabetes during pregnancy [14]. Various sociodemographic factors, including living in rural areas [5], being illiterate [5], having low socioeconomic status [12], being victims of any domestic violence, and either physical, sexual, or mental abuse [15], have an effect on the mother's health. The birth weight of neonates is influenced by the mother’s biological and demographic status [5]. In addition to biological and demographic factors, maternal nutrition is a significant element that affects birth outcomes throughout pregnancy. Malnutrition during pregnancy significantly negatively impacts a child’s lifespan [16].

Several studies have been conducted in Bangladesh regarding LBW [5,17–19]. A study was conducted to identify the causes of LBW and estimate the occurrence of LBW infants using machine learning algorithms [20]. Another study was conducted to examine adverse maternal circumstances and determine their association with LBW among women of reproductive age and their offspring [21]. However, evidence regarding the district-level variation of the prevalence of LBW and inadequate dietary diversity of the maternal infant young child nutrition (MIYCN) programme in rural areas is limited. Also, most of those studies in Bangladesh explored the factors associated with LBW. There is a lack of evidence regarding the socioeconomic inequalities of LBW. Further, exploring the association between inadequate maternal dietary diversity and LBW in the programme areas is crucial for better implementation and planning of the evaluation programmes. Therefore, this study aimed to identify the district-level prevalence variation of LBW, socioeconomic inequalities of LBW, and the association between inadequate dietary diversity and LBW in the rural areas of Bangladesh.

## METHODS

### Study design

We analysed pooled data from five cross-sectional surveys conducted at the household level as part of the evaluation of the MIYCN phase two programme implemented by BRAC, an international development organisation in Bangladesh.

### Study setting

These surveys took place from September 2016 to May 2018 in the rural areas of 26 districts and two urban slums of Dhaka, Bangladesh, included in the BRAC MIYCN programme coverage. The surveys were conducted in three phases during the same period at almost the same interval of the year as the previous surveys to minimise the effect of seasonality on the outcome indicators. In the MIYCN programme, we conducted eight surveys. But in this study, we included the last five surveys since the birth weight information was collected in these five surveys. The MIYCN survey timelines are presented in Figure S1 in the **Online Supplementary Document**.

### Sample size

The MIYCN programme’s primary outcome indicators were anaemia, micronutrient powder (MNP) coverage, and infant and young child feeding practices among children aged six to 59 months. Initially, we considered a 50% prevalence of MNP coverage, with a precision of 10% and a design effect of two. This led to a minimum estimated sample size of 192 households per district for caregivers of children aged six to 59 months across the first five cross-sectional surveys. Therefore, the total sample size for the first five surveys was 11 699. After conducting these five cross-sectional surveys, the study investigators, in agreement with donors, collaborators, and implementation partners, decided to plan the next three end-line surveys based on the findings from these five surveys. We again calculated the sample size considering a 10% effect size for anaemia, with 90% power, and a design effect of two for the remaining three surveys. The total sample size for these three surveys was 5237. Hence, the total sample size for the MIYCN programme from eight surveys was 16 936. For this article, we utilised pooled data from the last five surveys comprising 4651 mothers and children aged six to 59 months. We used sub-samples of 4651 among 10 198 since most caregivers didn’t recall their baby’s birth weight, and we excluded these samples from our analysis (Table S2 in the **Online Supplementary Document**).

### Sampling procedure

We applied a multi-stage sampling technique to select households at the community level. Initially, we utilised a systematic random sampling technique to choose primary sampling units (PSUs). To reach the minimum estimated sample size of 192 households in each district, we selected 16 PSUs from a predefined list sorted by district and sub-districts within those districts. In the second sampling stage, we employed a physical map-segment approach to divide the selected communities or PSUs. Finally, we adopted the expanded programme on immunisation method by rotating a bottle or pen placed at the centre of each segment. We then counted the households along the route and selected the fifth household. Data collection was facilitated using an Android-based tablet (smartphone) developed on the Open Data Kit platform (Get ODK, San Diego, California, USA). We applied the same sampling procedure for all the surveys.

### Outcome variable

The outcome variable in this study was LBW. According to the WHO’s definition, LBW is characterised by a birth weight <2500 g, while normal birth weight (NBW) is defined as a weight ≥2500 g [22]. We dichotomised LBW with ‘yes’ denoting LBW (assigned a value of one) and ‘no’ denoting NBW (assigned a value of zero). The birth weight information was collected based on maternal recall or from the health card of the children.

### Exposure variable

The exposure variable was the dietary diversity of the women. The dietary diversity scores (DDSs) were calculated based on the food groups of foods of seven diversified groups. The seven food groups included grains, white roots and tubers, plantains (group one), pulses (group two), dairy (group three), meat, poultry and fish (group four), eggs (group five), vitamin-A-rich fruits and vegetable (group six), and other fruits (group seven). The DDS ranged between zero to seven. The higher the DDS, the higher the food diversities. The dietary diversity was categorised into inadequate and adequate based on the median dietary diversity score [23]. The median DDS was four. The values of DDS from zero to four were considered inadequate dietary diversity, while scores ≥5 were considered adequate dietary diversity (Table S1 in the **Online Supplementary Document**).

### Independent variables

The independent variables were selected based on the literature review [12,13,24] and the availability of information in our dataset. The independent variables included the child’s age (continuous), child’s sex (male and female), mother’s age (categorised as ≤19 years and >19 years), and wealth index (categorised as poorest, poorer, middle, richer, and richest). The wealth index was calculated based on household materials (e.g. materials used for the floor, roof, and wall of the house), type of latrine used, and drinking water sources by performing a principal component analysis. Since we aimed to assess the relationship between maternal dietary diversity and LBW, we included a few important independent variables to assess the relationship controlling these important independent variables. Included more independent variables could potentially distort the true relation between maternal dietary diversity and LBW.

### Statistical analysis

The statistical analysis was performed using Stata, version 15.0 (Stata Corporation, College Station, Texas, USA). The analysis included both descriptive and analytical techniques. In descriptive statistics, categorical variables were summarised using frequencies and percentages, while continuous variables were expressed as means and standard deviations. The difference in the prevalence of LBW between the poorest and richest was assessed using the Pearson chi-squared test. To assess the socioeconomic inequalities in LBW prevalence based on asset-based socioeconomic status, we computed the concentration index (CIX). This index, derived from the Lorenz curve, measures the extent of inequalities, typically ranging from minus one to one. A positive CIX value suggests a higher prevalence of LBW among more affluent individuals. In comparison, a negative value indicates a higher prevalence of LBW among less affluent individuals, with a value of zero indicating no socioeconomic inequalities. We calculated both the absolute and relative measures of the socioeconomic inequalities of LBW. The absolute measure was calculated by subtracting the prevalence of LBW of the poorest group from the prevalence of LBW of the richest group. The relative measure of LBW was calculated by taking the ratio of the prevalence of LBW in the richest group to the poorest group. Additionally, a multivariable binary logistic regression model was performed to examine the relationship between LBW and maternal dietary diversity, adjusting for other independent variables and accounting for clustering effects. Interaction effects between mother age and dietary diversity were assessed to determine their combined influence on LBW. The regression analysis results were presented as adjusted odds ratios (AOR) with 95% confidence intervals (CIs). Statistical significance was determined by a *P*-value <0.05 and a 95% CI. The goodness of fitting of the models was evaluated using the Hosmer Lemeshow test. In the test, we found that *P* = 0.090 indicates that the model was fitted approximately well. We followed the Strengthening the Reporting of Observational Studies in Epidemiology (STROBE) guidelines in designing and reporting this study (Table S2 in the **Online Supplementary Document**).

## RESULTS

### Sample characteristics

The average age of the children was 28.6 months, while the average age of the mothers was 25.9 years. Among the children, 54.1% were male, and 7.9% of the mothers were adolescents. Regarding the wealth index, 16.9% of households were in the poorest group, whereas 27.4% were in the richest group (**Table 1**).

**Table 1:** Characteristics of the study population (n = 4651)

Variables	N (%)
Child’s age in months, x̄ (SD)	28.6 (14.4)
*6–23*	1933 (41.6)
*24–59*	2718 (58.4)
Child’s sex	
*Male*	2514 (54.1)
*Female*	2137 (45.9)
Birth weight	
*Normal (≥2500 g)*	4025 (86.5)
*LBW (<2500 g)*	626 (13.5)
Mother’s age in years, x̄ (SD)	25.9 (5.6)
*≤19*	368 (7.9)
*>19*	4283 (92.1)
Wealth index	
*Poorest*	787 (16.9)
*Poorer*	893 (19.2)
*Middle*	847 (18.2)
*Richer*	849 (18.3)
*Richest*	1275 (27.4)

LBW – low birth weight, SD – standard deviation, x̄ – mean

### Geographical variation of LBW

**Figure 1:**
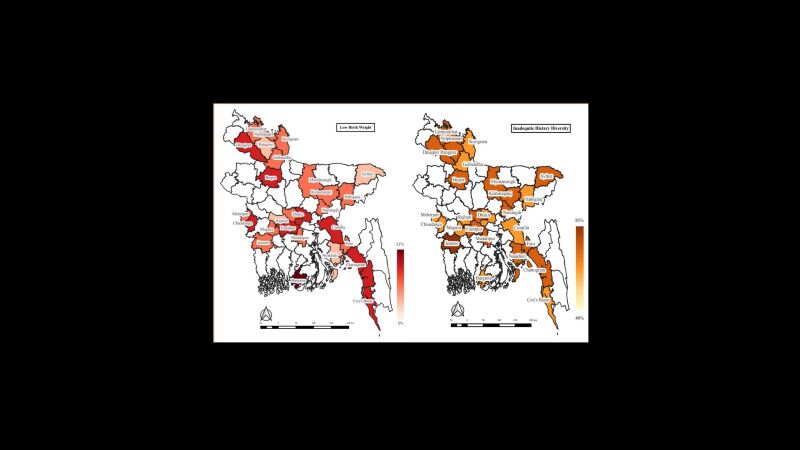
Geographical variations of low birth weight and inadequate dietary diversity.

**Figure 2:**
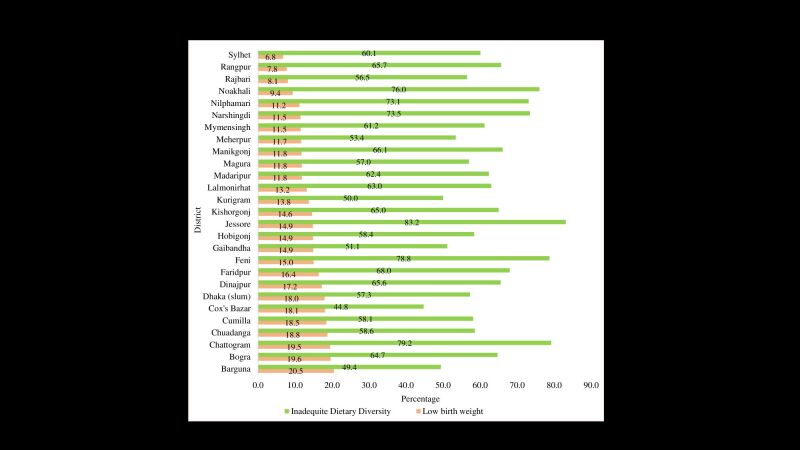
District-level variations of low birth weight and inadequate dietary diversity.

The prevalence of LBW was 13.5% (**Table 1**). The highly prevalent zone of LBW was the southern part of Bangladesh (**Figure 1**). Notably, a higher prevalence of LBW was identified in Barguna (20.5%), Bogra (19.6%), Chattogram (19.5%), Chuadanga (18.8%), Dinajpur (17.2%), Faridpur (16.4%), and Feni (15.0). Conversely, the prevalence of LBW in other districts remained below (15%), with the lowest rate recorded in Sylhet (6.8%) district (**Figure 2**).

### Geographical variation of inadequate dietary diversity

**Figure 3:**
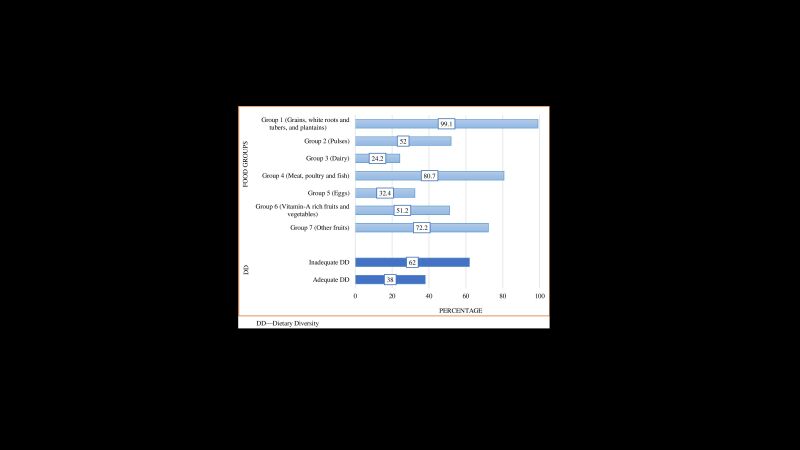
Distribution of different food groups and dietary diversity.

Among the food groups, most of the mothers (99.2%) consumed food from group one (grains, white roots and tubers, and plantains), whereas the lowest (24.2%) consumption group was group three (dairy). 62% of mothers had inadequate dietary diversity (**Figure 3**). The inadequate dietary diversity was highly concentrated in northern Bangladesh (**Figure 1**). Similarly, the higher prevalence of inadequate dietary diversity among women was observed in Jessore (83.2%), Chattogram (79.2%), Feni (78.8%), Noakhali (76.0%), Narshingdi (73.5%), Nilphamari (73.1%), etc. (**Figure 2**).

### Socioeconomic inequalities of LBW in rural Bangladesh

**Figure 4:**
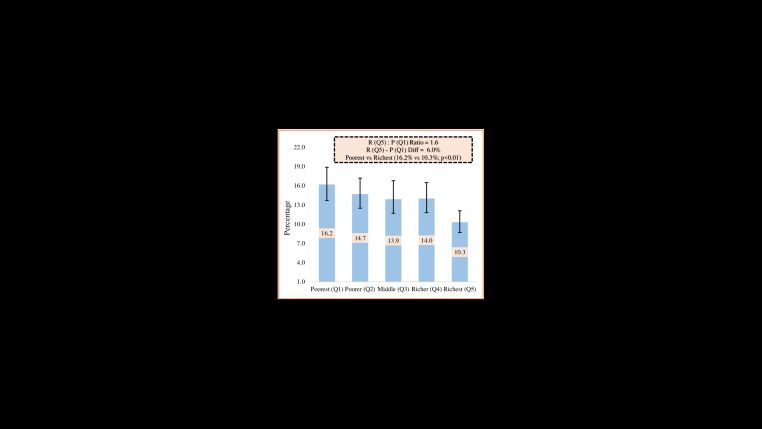
Socioeconomic inequalities of low birth weight in rural Bangladesh. R = Richest, P = Poorest, Q1 = First quintile, Q5 = Fifth quintile.

**Figure 5:**
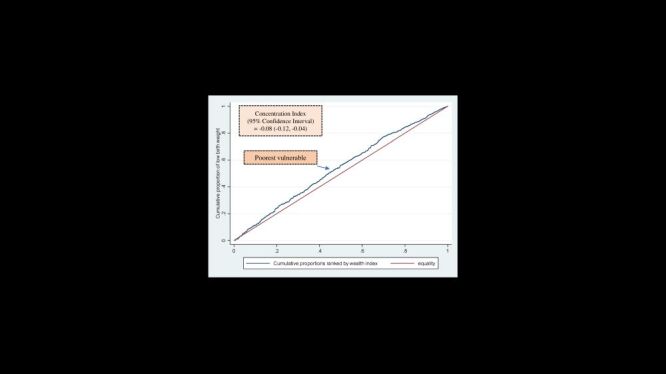
Concentration curve to measure the wealth-based inequalities of low birth weight.

The absolute difference in the prevalence of undernutrition between the poorest and richest groups was 6.0% (95% CI = 5.0, 6.8). Additionally, we identified a 1.6 ratio of LBW prevalence between the poorest (quintile one) and richest (quintile five) groups (**Figure 4**). Notably, the distribution of LBW prevalence was found to be disproportionate among socioeconomically disadvantaged groups, as indicated by CIX = –0.08 (95% CI = –0.12, –0.04) (**Figure 5**).

### Association between maternal inadequate dietary diversity and LBW

**Figure 6:**
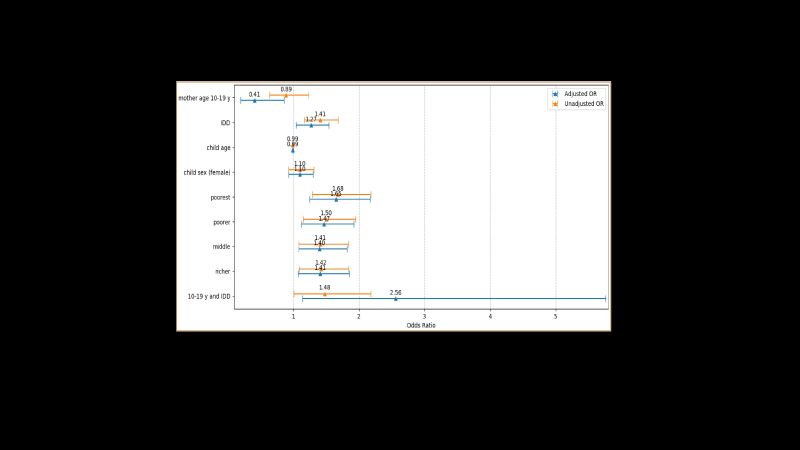
Association between maternal inadequate dietary diversity and low birth weight. 10–19 years and inadequate dietary diversity (IDD) is the interaction term between women aged 10–19 years and IDD. The reference category was women aged more than 19 years and adequate dietary diversity.

In the regression model, after adjusting potential confounders, we observed that women with inadequate dietary diversity had a 27% higher likelihood of giving birth to an LBW baby (AOR = 1.27; 95% CI = 1.04, 1.54). Additionally, women from the poorest, poorer, middle, and richer families presented increased odds (poorest: AOR = 1.65, 95% CI = 1.25, 2.17; poorer: AOR = 1.47, 95% CI = 1.12, 1.92; middle: AOR = 1.40, 95% CI = 1.08, 1.82; richer: AOR = 1.41, 95% CI = 1.07, 1.85) of delivering LBW babies compared to those from the richest families. Surprisingly, we found that adolescent mothers with inadequate dietary diversity had 2.56 times (AOR = 2.56; 95% CI = 1.14, 5.76) odds of giving birth to LBW babies compared to mothers who were adults and had adequate dietary diversity (**Figure 6**).

## DISCUSSION

This study aimed to explore the geographical variation, socioeconomic inequalities of LBW, and the association of LBW with maternal dietary diversity. We observed a high prevalence of LBW in the southern parts of Bangladesh. The richest-poorest inequalities of LBW exist in the rural areas of Bangladesh, where the MIYCN programme has been implemented. We also investigated a strong association between maternal inadequate dietary diversity and LBW. Notably, we found that adolescent mothers with inadequate dietary diversity were more likely to have LBW babies.

We found a high prevalence (ranging from 18.1 to 20.5) of LBW in the southern regions of Bangladesh, mostly in Chattogram, Cumilla, Barguna, and Cox’s Bazar districts. Consistent with our study findings, studies showed that the Chattogram regions had a higher likelihood of having LBW babies than the Khulna and Barisal regions [17,20]. A considerable regional variation in the prevalence of LBW was also reported in the prior study, with the highest rate in the Rangpur (28%) and the lowest rate in the Rajshahi (11%) [5]. Food insecurity could be one of the reasons for the high prevalence of LBW in the southern part. Households in the coastal belt, eastern hills, and haor experience higher levels of food insecurity during the rainy monsoon season [25]. In contrast, households in the northern chars and northwest experience lower levels of food insecurity during that time of year [25]. Extreme weather events have also resulted in considerable salinity in the coastal area and saline water mixing with cultivated lands. This leads to the abandonment of farmed land and reduces crop production [26]. Maternal and child undernutrition in Bangladesh has also been linked to these seasonal nutritional stresses in southern regions brought on by household-level food insecurity [27]. Along with food insecurity, the regional variation of LBW could be due to the improper allocation of resources in the country's existing maternity and childcare services, as well as a lack of understanding of the implications of LBW among residents of those regions.

This study identified the richest-poorest inequalities in the prevalence of LBW. The findings reveal higher LBW prevalence among the poorest compared to the richest, which is also in line with the prior studies [5]. Poverty has a direct impact on the birth weight of children in rural areas of Bangladesh due to factors like inadequate food intake, unclean housing, poor sanitation, a lack of capacity to seek medical attention, purchase of necessary medications, food, or hygiene items, and travel distance to health centres [28]. However, a study conducted in India revealed that socioeconomic status was a significant predictor of LBW in rural and urban areas [29]. Similar patterns have been found in the studies conducted in Bangladesh, Ethiopia, Sri Lanka, and sub-Saharan Africa, with low-wealth index households being one of the most prevalent factors causing LBW in urban and rural settings [19,30–32]. In contrast to our study finding, an earlier study conducted in rural Bangladesh found no correlation between LBW and economic status [28]. Considering this study was conducted in a rural area with antenatal services provided by a non-governmental organisation, its limitations may account for the reason its findings differ from those of previous research.

We found the likelihood of having LBW children was higher among mothers with inadequate dietary diversity. This result is in line with the research carried out in India [33,34] and Ghana [35]. Similarly, Zerfu et al. [36] observed that Ethiopian mothers with inadequate diets had a twofold higher risk of LBW (adjusted risk ratio (ARR) = 2.06; 95% CI = 1.03, 4.11) compared to mothers with adequate diets. This could be a result of the adequate diet during pregnancy, potentially including several essential nutrients required for the foetus and newborn’s growth and development. Previous epidemiological studies also showed that improved birth outcomes, like the birth weight of the children, were linked with better dietary practices during pregnancy [37]. Contrary to our findings, a recent randomised controlled trial in India reported that increased intake of dairy products, fruits, and vegetables before and during pregnancy through a formulated meal had no significant effect on birth weight [38]. Similarly, another study among the multi-ethnic Asian population showed no correlation between the weight of the child at delivery and the mother's intake of macronutrients during pregnancy [39]. The differences observed in these studies can be attributed to variations in research methodologies, sample sizes, socioeconomic backgrounds, and study designs.

A notable finding in our study was that adolescent mothers with inadequate dietary diversity had a higher likelihood of experiencing LBW babies. A Brazilian study of pregnant adolescents found that the likelihood of LBW increased by 3.5 times (adjusted prevalence ratio (APR) = 3.5; 95% CI = 1.49, 8.4) in the absence of nutritional care [40]. Likewise, prior studies observed that adolescents are at risk of not gaining enough weight during pregnancy and, subsequently, having an LBW child [41,42]. In cases where the adolescent mother's nutritional state is depleted, the partitioning would favour the mother over the foetus, resulting in several negative pregnancy outcomes, including LBW [43].

Efforts to minimise the geographical variations and inequalities of LBW and reduce the prevalence of LBW may entail a commitment to effective policy initiatives and interventions. To minimise the geographical variations and socioeconomic inequalities of LBW, it would be effective to allocate resources to the vulnerable districts and among the poorest households. Additionally, enhancing maternity and child healthcare services in these vulnerable districts could play a significant role in minimising geographical variations. The LBW seemed reduced by improving the nutritional status of women. Pregnant women in Bangladesh receive their nutrition knowledge primarily through antenatal care (ANC) [44]. A community-based balanced plate nutrition education intervention delivered by community health workers through ANC visits can successfully increase birth weight and promote dietary diversity in rural Bangladeshi women, especially among adolescent mothers [45]. Further, providing adequate nutrition for adolescent girls, supplementing iron and folic acid to women and adolescent mothers, providing balanced protein-energy supplements, and providing daily calcium supplementation at the regional and community levels can all be recommended to lower the prevalence of LBW [5]. Through the provision of nutrition services, case management, and support, home visits by community health workers may help expectant adolescents or low-income mothers achieve better delivery outcomes, like a lower likelihood of LBW [46]. Also, improving the financial situation of low-income households, such as by offering unconditional or conditional cash transfers, can lessen the incidence of LBW by reducing the likelihood of having a child with LBW in rural areas [47,48]. Policy and practice may take into account the present evidence, particularly increasing age at first marriage. Interventions beyond the home visits by health workers and four or more ANC visits during pregnancy might play a role in the prevention of LBW [49]. Evidence shows that high-risk women's likelihood of LBW delivery may be decreased by integrated community care coordination combined with outcome monitoring and reward for results [50]. The present study findings advance understanding regarding the prevalence of LBW and its associated factors, which may assist developers, hospital executives, and healthcare professionals in creating and carrying out effective clinical and public health initiatives aiming at reducing LBW in rural regions in Bangladesh. However, future studies including the case-control and cohort studies can be designed to establish the robust association between inadequate dietary diversity and LBW.

### Strengths and limitations

This study has several strengths. Firstly, it offers valuable insights into the current prevalence of LBW at the national level, utilising representative samples gathered from rural districts across the country. Secondly, the district-level LBW estimates present a valuable resource for government, policymakers, and non-governmental organisations, helping in the proper allocation of resources and targeted intervention programmes. This targeted approach allows for a more strategic distribution of resources, focusing on the most vulnerable districts. Third, the evidence of association of adolescent mothers with inadequate dietary diversity and a high chance of LBW will focus the policy makers for initiating ensuring diversified foods among adolescent mothers during their pregnancy. Fourthly, the robustness and representativeness of the estimates are enhanced by the large sample size and random sampling technique employed, covering various regions of Bangladesh.

However, there are certain limitations to acknowledge. This study utilised sub-samples to calculate LBW, thereby reducing the minimum estimated sample size. Additionally, data collection was limited to 26 out of 64 districts in rural Bangladesh, which may lack the representativeness of the whole country. Although data from two urban slums in Dhaka were included, these statistics may not accurately portray the reality of the urban LBW scenario. Consequently, further research is needed to estimate LBW in urban settings. The calculation of dietary diversity was based on the median value of seven food groups instead of ten food groups due to the merge of three groups among the three similar groups, which deviates from the calculations according to the Food and Nutrition Technical Assistance guidelines. Finally, the reliance on maternal recall for some infants' birth weight introduces the potential for recall bias.

## CONCLUSIONS

Geographical variations exist in LBW and maternal inadequate dietary diversity. The riches-poorest inequality of the prevalence of LBW exists in rural areas. Mothers with inadequate dietary diversity had a higher likelihood of giving birth to LBW neonates. Notably, adolescent mothers with inadequate dietary diversity had a higher chance of experiencing LBW babies than adult mothers with adequate dietary diversity. Based on our findings, we recommend that developing intervention strategies for reducing LBW should be targeted at the poorest households in rural areas. Interventions for improving the dietary diversity of adolescent pregnant mothers in the poorest households are expected to be worthwhile. The findings of our research will be influential in initiating long-term policies for reducing LBW and improving dietary diversity in the most vulnerable districts where the prevalence of LBW and inadequate dietary diversity is higher. Additionally, understanding the association between inadequate dietary diversity and LBW will help policymakers design interventions aimed at improving dietary diversity among pregnant women, with a particular focus on adolescent pregnant women, to reduce the prevalence of LBW.

## Additional material


Online Supplementary Document
