# Supplementary material for: Geographical variation, socioeconomic inequalities of low birth weight, and its relationship with maternal dietary diversity: Insights from the maternal infant and young child nutrition programme in Bangladesh
Source: J Glob Health. 2024 Oct 11;14:04209. doi: 10.7189/jogh.14.04209 (PMC11467772; doi:10.7189/jogh.14.04209)
Supplement: Online Supplementary Document [file jogh-14-04209-s001.pdf]

Table S1. Details description of seven food groups

Group 1	Grains, white roots and tubers, and plantains	Any food made from grain such as millet, wheat, rice, maize, semolina, atta=flour, noodles, porridge, jau, black cumin Any food made from roots or tubers such as alo, mula, white potatoes, white sweet potato, onions, garlic, ginger, kachu, shalgom, wol
Group 2	Pulses (beans, peas, lentils)	Any food made from lentils, beans, Bengal gram, channa, soya bean, ker, cowpea, sattu, peanut paste, kichuri, nuts, or seeds, dal
Group 3	Dairy products (milk, infant formula, yogurt, cheese)	Tinned, powdered or fresh milk (excluding breast milk), Cares or infant formula such as Cerelac, Lactogen, Mother Dairy, Nan Pro (Nestlé) Curd, lassi, yoghurt, chaach, cheese, paneer, mawa barfi, shrikhand, chanar sandesh or other milk products
Group 4	Meat, poultry and fish	Liver, kidney, heart, or other organ meats Any meat such as chicken, goat, sheep, beef, Fresh or dried fish, or seafood
Group 5	Eggs	Eggs
Group 6	Vitamin-A rich fruits and vegetables	Any food made from vegetables that have yellow or orange flesh such as red carrots, kachar, red sweet potatoes, lalshak Any dark green leafy vegetables such as palak, puishak, mula shak, pat shak, sarso, spring onion and radish leaves, coriander
Group 7	Other fruits	Any other fruits or vegetables such as coconut, apple, green mango, banana, pomegranate, lemon, guava, brinjal, tomatoes, beetroot, , cauliflower, cabbage, cucumbers, sweet corn, kudu, jack fruit, pineapple, pawpaw, water lemon, orange, lychee, plum, guava, black berries, dates, palms, dalims, kamranga, jambura, latkon, bel, ripe mangoes, ripe papaya

Table S2. STROBE Statement—Checklist of items that should be included in reports of ***cross-sectional studies***

	Item No	Recommendation	Page No
**Title and abstract**	1	(*a*) Indicate the study's design with a commonly used term in the title or the abstract	1
		(*b*) Provide in the abstract an informative and balanced summary of what was done and what was found	2
**Introduction**			

Background/rationale	2	Explain the scientific background and rationale for the investigation being reported	3-4
Objectives	3	State specific objectives, including any prespecified hypotheses	3-4
**Methods**			
Study design	4	Present key elements of study design early in the paper	4
Setting	5	Describe the setting, locations, and relevant dates, including periods of recruitment, exposure, follow-up, and data collection	4
Participants	6	(*a*) Give the eligibility criteria, and the sources and methods of selection of participants	4
Variables	7	Clearly define all outcomes, exposures, predictors, potential confounders, and effect modifiers. Give diagnostic criteria, if applicable	5
Data sources/ measurement	8*	For each variable of interest, give sources of data and details of methods of assessment (measurement). Describe comparability of assessment methods if there is more than one group	*4*
Bias	9	Describe any efforts to address potential sources of bias	N/A
Study size	10	Explain how the study size was arrived at	4
Quantitative variables	11	Explain how quantitative variables were handled in the analyses. If applicable, describe which groupings were chosen and why	5
Statistical methods	12	(*a*) Describe all statistical methods, including those used to control for confounding	6
		(*b*) Describe any methods used to examine subgroups and interactions	6
		(*c*) Explain how missing data were addressed	N/A
		(*d*) If applicable, describe analytical methods taking account of sampling strategy	
		(*e*) Describe any sensitivity analyses	N/A
**Results**			
Participants	13*	(a) Report numbers of individuals at each stage of study—eg numbers potentially eligible, examined for eligibility, confirmed eligible, included in the study, completing follow-up, and analysed	7
		(b) Give reasons for non-participation at each stage	N/A
		(c) Consider use of a flow diagram	N/A
Descriptive data	14*	(a) Give characteristics of study participants (eg demographic, clinical, social) and information on exposures and potential confounders	7

		(b) Indicate number of participants with missing data for each variable of interest	7
Outcome data	15*	Report numbers of outcome events or summary measures	5
Main results	16	(*a*) Give unadjusted estimates and, if applicable, confounder-adjusted estimates and their precision (eg, 95% confidence interval). Make clear which confounders were adjusted for and why they were included	7-10
		(*b*) Report category boundaries when continuous variables were categorized	5
		(*c*) If relevant, consider translating estimates of relative risk into absolute risk for a meaningful time period	N/A
Other analyses	17	Report other analyses done—eg analyses of subgroups and interactions, and sensitivity analyses	6
**Discussion**			
Key results	18	Summarise key results with reference to study objectives	12
Limitations	19	Discuss limitations of the study, taking into account sources of potential bias or imprecision. Discuss both direction and magnitude of any potential bias	14
Interpretation	20	Give a cautious overall interpretation of results considering objectives, limitations, multiplicity of analyses, results from similar studies, and other relevant evidence	12-14
Generalisability	21	Discuss the generalisability (external validity) of the study results	14-15
**Other information**			
Funding	22	Give the source of funding and the role of the funders for the present study and, if applicable, for the original study on which the present article is based	14

*Give information separately for exposed and unexposed groups.

Note: An Explanation and Elaboration article discusses each checklist item and gives methodological background and published examples of transparent reporting. The STROBE checklist is best used in conjunction with this article (freely available on the Web sites of PLoS Medicine at http://www.plosmedicine.org/, Annals of Internal Medicine at http://www.annals.org/, and Epidemiology at http://www.epidem.com/). Information on the STROBE Initiative is available at www.strobe-statement.org.

Figure S1: Survey timelines

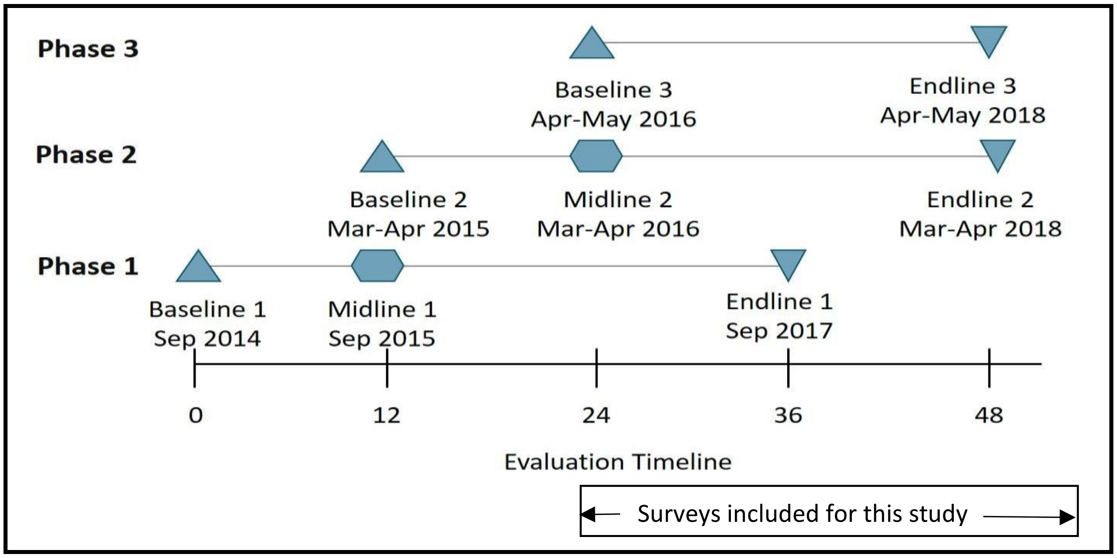

Sample size for first five surveys

For program course correction purposes the surveys aim to provide program coverage estimates, and anaemia prevalence estimates at the level of the district. This allows the research team to provide BRAC with district level data for the respective program areas. However, for assessing the overall coverage of the program, and overall anaemia prevalence, the full sample (i.e. sum of all districts) was used.

The following formula was used to calculate sample size for the coverage survey:

$$n = \frac{Z_{\alpha}^{2}\ P(1-P)}{d^{2}}\ x\ DE$$

Where, n = required sample size for each survey, expressed as number of units of analysis,

P: expected program coverage,

d: error (level of precision),

Z_{α}: value at 95% confidence level

DE =design effect

For the district level estimates, the following are assumed: 50% estimated prevalence, precision of +/- 10%, Z_{α} of 1.96 and a design effect of 2. This yields minimum sample size of 192 households per district.

Table: Estimated sample size for first five surveys

Survey number	Survey time point	Number of districts to be surveyed

Number of districts rolled out by BRAC		Baseline	Midline		Sample size (HH) estimated	# of HH surveyed
10	1	X		10	1920	1927
	2		X	10	1920	1924
15	3	X		15	2880	2887
	4		X	15	2880	2883
09	5	X		09	1728	2078*
**Total**						**11699**

*350 HH surveyed from two urban slums (Ashrafabad and Manda) at Dhaka

Sample Size for last three surveys

After conducting these five cross-sectional surveys, the study investigators, in agreement with donors, collaborators, and implementation partners decided to plan the next three endline surveys based on the findings from these five surveys.

If we considered the prevalence of anemia, good IYCF practice and effective coverage of Pushtikona-5 before and after the programme (expected), then, we used the following formula to calculate estimated sample size

$$n = \frac{P_1\ (100 - P_1) +\ P_2\ (100 - P_2)}{(P_2 - P_1)^2}\ X\ \ (Z_\alpha\ +\ Z_\beta\ )^2\ \ X\ DE$$

n= desired sample size
P_1= (Average) Prevalence of anemia/ percentage of IYCF practice/Effective coverage of Pushtikona-5 in the last five surveys
P_2= Expected prevalence of anemia/ IYCF practice/Effective coverage of Pushtikona-5 after the intervention
Z_α = Value at 95% CI = 1.96
Z_β = Value if power of test is considered 90% = 1.282
DE=Design Effect=2

According to this formula the required sample size for following estimations were-

Serial number.	Estimate being considered	P_1 (%)	P_2 (%)	n= HHs to be surveyed per platform
1	Avg. prevalence of anemia from last 5 surveys	42	32	969
2	Avg. percentage of IYCF practice from last 5 surveys	36	50	515
3	Avg. percentage of effective coverage from last 5 surveys	3	8	864

For P, we have considered average good IYCF practice percentage, average anemia prevalence and average effective coverage of Pushtikona-5 from the previous surveys (3 baselines and 2 endlines). But

the average anemia prevalence yields the highest sample size i.e. highest number of HHs per platform, so we can assume that it can represent the other prevalence, too (mentioned above). In total, in three phases (at three different platforms) the required sample size=969X3=2907. In each platform of BRAC programme 969 households were distributed accordingly in the districts under the platforms.

In first phase (at MNCH platform), there are 10 districts, so for each district the number of households were surveyed=969/10=96.9 ~97. We selected 16 PSUs (communities where BRAC's MIYCN programme is being implemented) from each district from the list provided by BRAC by simple random sampling and thus 97/16=6.06~7 households were selected from each PSU. In this way, the estimated total sample size in MNCH area was (7X16X10) =1120, since there are 10 districts in MNCH platform. Similar procedure was applied for estimating sample size from other two platforms. Therefore, the estimated minimum sample size per platform was as follows:

Platform	# of district/platform	# of hhs (considering 16 PSUs) per PSU	Total sample size per platform
MNCH	10	(969/10)/16=6.06,~7	7X10X16=1120
Alive and Thrive	15	(969/15)/16=4.04, ~5	5X16X15=1200
Nutrition	9	(969/9)/16=6.73, ~7	7X16X9=1008
Total sample (in 3 phases)			3328

So, around 3,500 households were sampled.

Additional samples for high performing areas

Because a secondary objective of this overall endline surveys was to quantify the programme effect on the participants who are exposed to high function of the programme, oversampling was effective to create a second, per protocol analytic sample. This was achieved by selecting an additional sample of households in high-functioning areas. Because, this is a more exploratory analysis for which estimates of heterogeneity/variance are unknown, meaningful sample size estimates are difficult. We used information from BRAC to quantify a meaningful and feasible additional sample. BRAC monitoring estimates suggest that 40% of SS are highly functional (based on program performance criteria), suggesting a similar likelihood of selecting a high functioning PSU in each district. We selected an additional sample in each of the three phases that is approximately 40% of the full sample for these per protocol analyses, translating to 500 households in each platform. This estimation is based on feasibility considerations. Consequently, the total sample size was 5,200. The distribution of sample across the platforms was as follows:

**Platform**	# of District/ Platform	**For Usual PSUs**		**For High Performing PSUs**			
		# of PSU	# of HH per platform	# of PSU	# of HH per platform	Total # of HH for both area	Total HH surveyed
MNCH	10	10x16=160	160x7=1120	10x6=60	60x7=420	1120+420=1540	1540
Alive and Thrive (A &T)	15	15x16=240	240x7=1680	15x4.5=67.5~68	68x7=476	1680+476=2156	2310
Nutrition	9	9x16=144	144x7=1008	9x4.5=40.5~41	41x7=287	1008+287=1295	1387
**Total**	**34***	**=544**	**=3808**	**=169**	**=1183**	**=4991**	**5237**

*8 districts were common for both A&T and Nutrition platforms
